# Knowledge mobilization with and for equity-deserving communities invested in research: A scoping review protocol

**DOI:** 10.1371/journal.pone.0310660

**Published:** 2025-11-21

**Authors:** Ramy Barhouche, Samson Tse, Fiona Inglis, Debbie Chaves, Erin Allison, Tina Colaco, Melody E. Morton Ninomiya

**Affiliations:** 1 Department of Psychology, Wilfrid Laurier University, Waterloo, Ontario, Canada; 2 Library, Wilfrid Laurier University, Waterloo, Ontario, Canada; 3 Department of Health Sciences, Wilfrid Laurier University, Waterloo, Ontario, Canada; PLOS: Public Library of Science, UNITED KINGDOM OF GREAT BRITAIN AND NORTHERN IRELAND

## Abstract

The practice of putting research into action is known by various names, depending on disciplinary norms. Knowledge mobilization, translation, and transfer (collectively referred to as K*) are three common terminologies used in research literature. Knowledge-to-action opportunities and gaps in academic research often remain obscure to non-academic community partners and researchers in communities, policy and decision makers, and practitioners who could benefit from up-to-date information on health and wellbeing. Academic research training, funding, and performance metrics rarely prioritize or address non-academic community needs from research. We propose to conduct a scoping review on reported K* in community-driven research contexts, examining the governance, processes, methods, and benefits of K*, and mapping who, what, where, and when K* terminology is used. This protocol paper outlines our approach to gathering, screening, analyzing, and reporting on available published literature from four databases.

## Introduction

Bridging the gap between produced research knowledge and action, policy, and/or practice has been a common goal across disciplines for the past few decades, often involving synthesis, analysis, dissemination, and application [1]. However, there has been some confusion in the use of various terms and definitions to represent this goal and its application process. In academia, for example, terms such as *knowledge translation*, *knowledge mobilization*, and *knowledge transfer* are widely utilized [2]. Similarly, Canada’s *Tri-Agencies*, the three main federal funding agencies for research, employ varied terminology and definitions for disseminating research knowledge. For instance, the Social Sciences and Humanities Research Council (SSHRC) [3] defines knowledge mobilization as an umbrella term encompassing activities related to the production and use of research results, including synthesis, dissemination, transfer, exchange, and co-creation by researchers and knowledge users. The Canadian Institutes of Health Research (CIHR), on the other hand, use terms like knowledge translation, integrated knowledge translation (IKT), and end-of-grant knowledge translation, emphasizing a dynamic and iterative process of knowledge synthesis, dissemination, exchange, and ethically-sound application [4]. The manuscript will adopt the term K* to collectively refer to these terms [5].

There has been a recent trend for utilizing research findings to inform practices, policies, and decisions through K* efforts. However, there is still a significant gap in research application, and knowledge derived from research has not effectively translated into health practices and policies [6]. The gap between research findings and application can be attributed to sociocultural, organizational, and economic factors that play a significant role influencing the research and mobilization process [1,7]. Furthermore, academics may lack readiness to engage in K* work, or if they do, their existing heavy workload limits their capacity to do so [8]. This disconnect significantly impacts community members, who are often knowledge users.

Similarly, community-based participatory research (CBPR) is recognized as a shift in the research paradigm that can enhance the utilization of research knowledge by involving knowledge users more extensively in the research process [9]. Like K*, CBPR also includes different aspects of shared and active community engagement in the research process, as well as enhancing community health through the integration of research and action [10]. In particular, CBPR complements IKT by emphasizing a democratic process of co-creating knowledge that aligns closely with the needs of knowledge users [9]. Thus, CBPR is essential for research that aims to enhance the health and wellbeing of individuals who are systematically marginalized or discriminated against. Traditional research has often been characterized by an unequal power dynamic between researchers and participants, a gap that CBPR can address [11]. For example, Christensen [12] and Morton Ninomiya et al. [13] have demonstrated the effectiveness of CBPR in engaging Indigenous communities in a respectful and culturally responsive way that led to impactful research outcomes. The essence of K* lies in the significance it brings to research. To promote equity and sovereignty-deserving communities, it can be argued that it is ethically imperative to ensure that community-driven K* needs are integrated into CBPR.

## Objectives

With the diverse terminology and definitions used to describe the “knowledge-to-action” gap, there is understandable confusion and potential frustration regarding the operationalization and use of K* terms. A preliminary search of databases revealed no current or ongoing scoping reviews on this topic. The involvement of community members and partners from equity seeking, deserving, or denied groups is essential for research committed to 1) addressing health and wellness inequities faced by different communities and/or 2) “nothing about us without us”. Similarly, we propose to conduct a scoping review study that is conducted by a team of scholars that are part of at least one equity seeking, deserving, or denied community themselves.

For the purposes of this scoping review, we define equity seeking, deserving, or denied groups as people who are structurally and systemically secluded by those in power – groups such as Indigenous communities; refugees, newcomers, and asylum seekers; settled racialized diasporas; two spirit, lesbian, gay, transgender, queer, intersex, asexual, and more (2SLGBTQIA+) communities; individuals with mental health challenges, disabilities, or chronic conditions; and older adults. We use the term ‘community partners’ to refer to non-academic members of equity seeking, deserving, or denied groups communities who are actively involved in the design, implementation, or dissemination of K* projects related to academic research. This includes, but is not limited to, community-based organizations, Indigenous knowledge keepers, community health workers, and individual community members.

## Research questions

The *scoping review* will answer the following questions about research aimed at supporting and serving non-academic equity and/or sovereignty-seeking/deserving/denied communities:

1. How are terms and concepts around knowledge mobilization (K*) being used, defined, and cited in published literature?
2. In what ways are community partners involved in K* priorities, planning, and efforts?
3. What are reported wellbeing-related impacts and outcomes being addressed within communities through K* efforts?

## Methods

### Search strategy

This scoping review protocol was developed using the JBI Manual for Evidence Synthesis [14] and adheres to the PRISMA-ScR as developed by Tricco et al. [15]. As a team, we identified 11 papers [16–26] that met all inclusion criteria to use as a test set while we developed our search strategy. After extensive testing, refining, and discussions as a team about concepts and terminology, the initial Medline (ProQuest) search strategy was finalized by two academic librarians [DC, FI]. This search was then translated by [RB] for PsycINFO (ProQuest), CINAHL with Full Text (EBSCOhost), and Web of Science Core Collection (Clarivate).

The search strategy focuses on four primary concepts: vulnerable populations (i.e., equity and/or sovereignty-seeking/deserving/denied communities), community-based research, K*, and health and wellbeing. The search terms around the concept of “vulnerable populations” were adapted from four published search strategies [27–29]. The terms around the concept of K* search strategy were adapted from Morton Ninomiya et al. [30]. We limited the search by date, including publications in 2010 onwards due to the increased international discourse and interest in K* in health and wellness research beginning at that time. Searching for research with equity and/or sovereignty-seeking/deserving/denied communities, often framed as “vulnerable populations”, is challenging because it sometimes includes offensive and outdated terms that are exclusionary. These terms were included in the search strategy to ensure a comprehensive search. The authors acknowledge the harmful nature of these terms and inform the reader of their inclusion. We will conduct a backwards and forwards search of the references in 1) review and discussion papers that do not meet the inclusion criteria, and 2) all included papers after full-text screening – in search of additional K* terms that we may have missed. See S1 Table for the full Medline search strategy.

All relevant data underlying the findings presented in this study will be made available upon study completion in the Open Science Framework (OSF) repository. We chose OSF because it is a free and open-access repository suitable for this type of data and commonly used in our field. The data will be available upon completion and a DOI will be assigned.

### Inclusion and exclusion criteria

The scoping review will only include primary studies published in English in which authors identify 1) their understanding of K* terminology with a definition, description, or citation; 2) *how* academic researchers engaged with non-academic community partners; 3) *what* K* efforts took place; and 4) *how* K* efforts impacted individual, family, and/or community wellbeing. We will exclude grey literature sources including reports, books, commentaries, and theses/dissertations from the scoping review. Because of the rise of international discourse and explicit interest in K* in health and wellness research that started around the 2010s, the date limit will be from 2010 to present. Only papers that are about equity and/or sovereignty seeking/deserving/denied communities will be included. The inclusion and exclusion criteria and their rationale are listed in Table 1 below.

**Table 1:** Inclusion/exclusion Criteria and Rationale.

Title and Abstract Screening Phase		
Include	Exclude	Rationale
Printed in English.	Printed in languages other than English.	It is the only shared fluent language of the research team.
Published in 2010 or later.	Published before 2010 or older.	We want the most updated, especially considering the rapid changes in KT literature. We are interested in seeing how it has been developed in the past 15 years or so. Google trends show consistent usage in search terms from 2010 and beyond.
Primary research papers, including case studies.	Discussion papers, autoethnographies, and reviews.	We want papers that are researching and applying K*.
Peer-reviewed and published.	Grey literature.	Interested in community-partnered academic research that often reflects disciplinary training, practice, language, and funding. For a global study, including grey literature would not be feasible.
Use specific terminology to describe K* in their paper.	Papers that do not use a specific term(s) to refer to K* initiatives, even if they describe elements of K*.	We are interested in terms people use to name and describe K* initiatives they facilitate.
The study addresses or supports something that aims to improve wellbeing for equity and/or sovereignty-deserving communities.	Exclude studies focused on capitalistic endeavors for monetary gain and power, or focused on communities that are not equity or sovereignty-deserving.	We are only interested in K* efforts on studies that aim to improve forms of wellbeing for communities who do not have equitable access to health and wellbeing.
**Full-text screening (added criteria)**		
Authors define or cite K* term(s) being used in the context of the study.	Papers that are unclear how the authors understand or use K* terminology.	This is an essential component of this study.
Authors state how non-academic community research partners were involved in the study, including K* related activities. Were involved in the study beyond being research participants.	Authors of the paper do not indicate how community was involved in the study, including K* related efforts.	Understanding how non-academic community partners are involved in the research and K* are key to this study.
Authors state impacts and outcomes from K* efforts, in relation to non-academic research partner needs and priorities.	Papers that do not identify impacts and outcomes from K* efforts in relation to non-academic research partner needs and priorities.	The impacts and outcomes from K* effort on non-academic researchers is the focus of the scoping review.

### Source of evidence selection

Search results will be imported into Covidence [31] and duplicate records will be removed automatically. To ensure high inter-rater reliability, four researchers [RB, JK, MM, ZP] involved in the screening process will screen the same 25 randomly selected records, based on the titles and abstracts. If we do not reach a minimum of 75% agreement, we will repeat the process with five more randomly selected records, until 75% agreement has been established.

Each title and abstract will be independently screened by two researchers. If one researcher thinks the record meets the inclusion criteria and the other does not, papers will be put in a “conflict” list. Each item in the conflict list will be resolved with a conversation between the two researchers and if consensus cannot be reached, the record will be included for full-text screening.

All papers included after the title and abstract screening will be uploaded for the full-text screening. Each full-text will be independently screened by two researchers and again, conflicts will be resolved through a conversation between the two researchers involved. If researchers cannot reach consensus, senior researcher [MMN] will make the final decision. Reasons for full-text exclusion will be tracked within Covidence and reported in the results. Only papers that met the inclusion criteria based on the full-text screening will be included for data extraction and analysis.

### Data extraction and analysis

#### Data extraction.

The scoping review extraction fields (Table 2) was developed by ST, RB, and MMN to record key K* information from the literature that will be selected, including author, reference, and relevant results aligned with the study’s research question [32]. Data from all included full-texts will be manually extracted and recorded in Covidence [31]. Two researchers will read through the K* literature and extract the data separately. The researchers will then meet to discuss and compare the extracted data to ensure accuracy and reliability.

**Table 2:** Data Extraction and Analysis.

Category	Description
General Data	• Publication Year • Location of study (region, country) • Location of lead/senior researcher affiliation (region, country) • Discipline (field of research and/or author affiliation)
Study	• Population studied/served/partnering • Research approach, methodology & methods • Research governance (e.g., role of community partner(s))
K*	• Authorship inclusion of community involved • K* terminology used • K* definition(s) and/or citations • Community partner involvement in K* prioritizing, planning, implementing • Other non-researcher rights/stakeholders involved in K* prioritizing, planning, implementing • Target group of K* • K* aims • Description of all K* efforts at planning & development stage • Description of all K* efforts at implementing stage
Outcomes	• Reported impacts or outcomes • Methods of tracking impacts and outcomes

#### Data analysis.

We will use thematic analysis as described by Braun and Clarke [33,34] to identify, analyze, and report on themes within the open-ended extracted data. The analysis of extracted open-ended and descriptive K* information and outcomes will be analyzed over six phases: [1] becoming familiar with the data, [2] generating initial codes, [3] searching for themes, [4] reviewing the themes, [5] defining and naming themes, and [6] producing the final report.

Once the extracted data is approved, the research team will each familiarize themselves with it and conduct an initial analysis independently before discussing and reaching a consensus on themes. After the consensus is reached, the team will re-analyze and revise the data results one more time before sharing them with the rest of the research team. Finally, in the sixth phase, the analysis findings will be incorporated into the results section, supported by quotations from the literature.

## Discussion

To our knowledge, this scoping review analysis is the first of its kind. Since 2010, there have been related reviews conducted on *integrated knowledge translation* with health policies [35,36] a realist evaluation of *knowledge transfer* implementation by health researchers [37]; *knowledge translation* and its supported definitions, theories, models, and frameworks [38]; and knowledge translation in Indigenous health research [39], for example. Our scoping review analysis will gather a broader disciplinary scope of literature and terminology than other reviews to date. Our scoping review is unique in that it will focus on studies where non-academic community partners are presented as being clearly invested and engaged in K* efforts – in ways that address inequities and support sovereignty, improving the health and wellness of equity-deserving/seeking/denied communities.

There is growing interest globally within academic and research institutions, research funding bodies, and government priorities to support research with equity-seeking/deserving/denied communities. Evidence to suggest the growing interest are reflected in university strategic plans, Truth and Reconciliation Calls to Action, targeted funding calls, and government public health frameworks, for example. Despite the increased attention to “applied research” with emancipatory goals, community-based researchers have been writing about limitations, challenges, and the inequitable nature of research funding timelines, accountabilities of researchers and funders to communities – for many years. Given that most scientific research is written, and therefore framed and curated, by academic researchers, it will be interesting to see how authors use K* terminology with vagueness to imply research “value” versus describing in compelling detail how community made informed decisions around K* priorities and activities.

We assert that research training programs and curriculum in disciplines invested in human health and wellbeing must educate, mentor, and model K*. That said, there is a dearth of training and research in the area of K* with equity or sovereignty-seeking/deserving/denied communities. The asymmetry between potential and actual transformation through research can be addressed, in part, by making exemplary K* research more visible and calling on authors of future papers study and report on how K* was planned, prioritized, implemented, and evidenced.

### Limitations

A limitation of the scoping review is the lack of uniform terminology on the topic of K*, across disciplines, communities, and geographies. As the topic of K* for research involving equity and sovereignty-seeking/deserving/denied communities remains unexplored, there are no consistent subject headings for the research team to reference or use as a guide for the literature searches. Our analysis will be limited by the keywords we have identified as relevant and introduces bias, as we elucidate additional K* terms being used. We aim to minimize this bias by conducting a backwards and forwards search of the references in review and discussion papers – in search of additional K* terms that we may have missed. In addition, limiting the scoping review to English could create a limitation to explore non-English literature, since this paper is aiming for a global understanding of these terms.

Our study only includes peer-reviewed published literature and excludes grey literature such as study reports, theses, and dissertations where K* may be defined, discussed, and described. We excluded grey literature for feasibility reasons only. Including it in our global and inter/multidisciplinary scoping review would be unwieldy. Creating a comprehensive and strategic grey literature search would necessitate limiting the search by geography, discipline, or date. Furthermore, given that K* language currently carries the most currency in academic research and funding cultures, we felt justified in prioritizing peer-reviewed literature for this scoping review. We acknowledge that excluding grey literature has limitations. Critically, by focusing solely on published academic literature, we may inadvertently overlook valuable K* efforts and knowledge generated within Indigenous and racialized communities that may not be found in many academic outlets. This exclusion risks perpetuating existing inequities by underrepresenting community-led initiatives and potentially reinforcing the idea that K* only “counts” if it is validated by academic publication. The findings from this scoping review will be used to inform future primary research studies on K*. The primary studies will reveal if and how published literature adequately accurately reports on effective and impactful K* in community-partnered research with equity- and sovereignty-deserving communities.

Moving forward, we recognize the importance of incorporating community-generated knowledge in our understanding of K*. Future research with a sharply focused K* angle should prioritize exploring the grey literature, including community reports, organizational publications, and other forms of community-led communication. Our future research program will prioritize direct engagement with equity- and sovereignty-deserving communities, ensuring that their perspectives and knowledge, regardless of publication status, are central to our understanding and implementation of K*. This scoping review, while focused on academic literature, will serve as a foundation for this future community-engaged work.

For the scoping review portion of our study, we will exclude papers that do not report on outcomes or impacts from K* efforts. Unless a study explicitly includes tracking, reporting, or observing outcomes and impacts from the K* efforts, authors may submit their manuscripts to journals before K* impacts and outcomes are known – and – the life cycle of most research grants are too short to include studying K* impacts and outcomes.

## Conclusion

The combination of using a scoping review to gather, examine, and visualize literature reporting on K* terminology, definitions, and priorities in research with equity or sovereignty-seeking/deserving/denied communities globally will paint an aerial view and nuanced summary of impactful research. Our study findings will be used to draw attention to examples of transformative research as well as reveal patterns of K* activities, outcomes, ethics, and accountabilities across geographies and disciplines.

## Supporting information

S1 Table Search Strategy. Search Strategy in Medline (ProQuest).
(DOCX)

S1 Checklist PRISMA-P checklist. (DOCX)
