# Supplementary material for: Knowledge mobilization with and for equity-deserving communities invested in research: A scoping review protocol
Source: PLoS One. 2025 Nov 21;20(11):e0310660. doi: 10.1371/journal.pone.0310660 (PMC12637959; doi:10.1371/journal.pone.0310660)
Supplement: S1 Table — Search Strategy in Medline (ProQuest). (DOCX) [file pone.0310660.s001.docx]

# Supplementary Information

S1 Table. This is The Search Strategy in Medline (ProQuest)

| S1 | ((MESH.EXACT("Mentally Ill Persons") OR MESH.EXACT("Drug Users") OR MESH.EXACT("Alcoholics")) OR tiab(mental* ill* OR "mental disorder" OR "mental disorders" OR PTSD OR "post traumatic stress" OR "posttraumatic stress" OR "drug user" OR "drug users" OR "drug dependence" OR "drug dependencies" OR "drug dependent" OR addict* OR alcoholi* OR pwud) OR tiab((drug OR substance*) NEAR/3 (abuse* OR use* OR illegal OR illicit OR addict*))) OR ((MESH.EXACT("Sex Workers") OR MESH.EXACT("Prisoners") OR MESH.EXACT("Pregnancy in Adolescence")) OR tiab((prostitute* OR "sex worker" OR "sex workers" OR "released prisoner" OR "released prisoners" OR "formerly incarcerated" OR "pregnant teen*" OR "pregnant youth*" OR runaway* OR "run away")) OR tiab((traffick* NEAR/3 (youth* OR women OR woman OR child* OR person* OR people*)))) OR ((MESH.EXACT("Ill-Housed Persons") OR MESH.EXACT("Homeless Youth") OR MESH.EXACT("Working Poor") OR MESH.EXACT.EXPLODE("Socioeconomic Factors")) OR tiab((poverty OR impoverished OR "working poor" OR unemploy* OR under-employed OR "transportation deficit*" OR "low soci* status" OR "low income*" OR low-SES OR hard-to-house OR homeless* OR under-housed OR underhoused OR "lack of housing" OR squatter* OR homeless* OR vagrant* OR indigent OR "hard to house" OR "inner city" OR "downtown core" OR "city core" OR "skid row" OR rural OR remote OR "low socioeconomic" OR "low SES" OR "poorest poor")) OR tiab((street NEAR/3 (worker* OR people OR child OR children OR youth))) OR tiab((hous* NEAR/3 (substandard OR insufficien* OR unstabl* OR under OR instabil*)))) OR ((MESH.EXACT("Child, Abandoned") OR MESH.EXACT("Enslaved Persons")) OR tiab(elders OR elderly OR oldest OR old OR senior citizen* OR shut-in OR house-bound OR neglected OR "older adult" OR "older adults" OR "battered women" OR "intimate partner violen*" OR "domestic violence") OR tiab((battered NEAR/3 (spouse* OR wife OR wives OR partner))) OR tiab((abuse* NEAR/3 (elder* OR child*)))) OR ((MESH.EXACT.EXPLODE("Sexual and Gender Minorities") OR MESH.EXACT("Bisexuality") OR MESH.EXACT("Transsexualism") OR MESH.EXACT.EXPLODE("Homosexuality")) OR tiab((LGBT* OR GLBT* OR 2SLGBT* OR 2SGLBT* OR lesbian* OR gay* OR bisexual* OR transgender* OR queer* OR "gender identit*" OR "gender minorit*" OR SGM OR GSM OR "gender nonconforming" OR "gender non-conforming" OR genderqueer OR "gender queer" OR "gender neutral" OR "gender fluid*" OR "gender variant" OR "mixed gender*" OR bigender* OR agender* OR pangender* OR "gender crossing" OR "sexual orientation*" OR pansexual* OR asexual* OR demisexual* OR bi-curious OR "sexual minorit*" OR nonbinary OR non-binary OR homosexual* OR trans-curious OR transcurious OR trans-sexual* OR transsexual* OR trans-people* OR transpeople* OR trans-person* OR transperson* OR trans-individual* OR transindividual* OR trans-woman OR transwoman OR trans-women OR transwomen OR trans-men OR transmen OR trans-man OR transman OR trans-girl* OR transgirl* OR trans-boy* OR transboy* OR trans-spectrum* OR transspectrum* OR "man loving man" OR "men loving men" OR "woman loving woman" OR "women loving women" OR "men who have sex with men" OR "women who have sex with women" OR F2M OR M2F OR "male to female" OR "female to male" OR "two spirit*" OR "2 spirit" OR TGNC OR "third gender"))) OR (MESH.EXACT.EXPLODE("Disabled Persons") OR tiab((blind OR visual* impair* OR hearing impair* OR deaf OR amputat* OR paraplegic* OR quadraplegic* OR wheelchair* OR disabled OR disabilit*) OR ("brain injured" OR "brain injuries" OR "brain injury" OR "brain damage" OR "brain damaged"))) OR (MESH.EXACT.EXPLODE("Indigenous Peoples") OR tiab((Metis OR Indigenous* OR Aboriginal* OR Amerindian* OR Autochtone* OR "First Nation*" OR Inuit OR Innu OR Inuk OR Inuvialuit OR tribal OR "first people*" OR "American Indian*" OR "native American*" OR "Alaska native*"))) OR ((MESH.EXACT("Ethnicity") OR MESH.EXACT("Transients and Migrants") OR MESH.EXACT("Refugees") OR MESH.EXACT("Cultural Diversity")) OR tiab((diversity OR minority OR minorities OR transient* OR migrant* OR immigrant* OR ethnic* OR racial* OR colonize* OR refugee* OR asylee* OR "war torn")) OR tiab((vulnerable OR migrant OR transient* OR marginal* OR "at risk" OR impoverished) NEAR/2 (population* OR people OR person* OR individual* OR child* OR youth* OR population* OR worker* OR men OR women OR man OR woman))) OR (MESH.EXACT("Vulnerable Populations") OR tiab((vulnerable OR equit* OR inequit* OR inequality OR equality OR disparit* OR discriminat* OR exclude* OR exclusion OR "high risk" OR underserved OR marginalised OR marginalized OR disadvantage* OR underprivileged OR underrepresented)) OR tiab((hard OR difficult) NEAR/2 (reach OR locate OR find OR treat OR engage))) |
| --- | --- |
| S2 | (MESH.EXACT("Community-Based Participatory Research") OR MESH.EXACT("Community Networks") OR MESH.EXACT("Community-Institutional Relations")) OR tiab((community OR civic) NEAR/3 (based OR participat* OR develop* OR uptake OR engage* OR partner* OR relation* OR driven OR involve* OR collaborat* OR impact OR exchange OR advocate*) OR non-profit OR stakeholder* OR rightsholder* OR "action research") OR (tiab("mode 2 research") OR tiab("engaged scholarship") OR tiab("integrated research") OR tiab("cultural broker*") OR tiab("knowledge broker*") OR tiab(co-production OR coproduction OR co-generation OR cogeneration) OR tiab(research NEAR/3 user) OR tiab(partner*)) |
| S3 | (MESH.EXACT("Translational Science, Biomedical") OR MESH.EXACT("Diffusion of Innovation") OR MESH.EXACT("Information Dissemination") OR MESH.EXACT("Implementation Science")) OR (tiab("diffusion of innovation") OR tiab("bench to bedside") OR tiab("implementation of existing research knowledge") OR tiab("linkage and exchange") OR tiab("knowledge to action") OR tiab("implementation science") OR tiab("know-do-gap")) OR tiab((knowledge OR evidence OR research OR information) NEAR/3 (transfer OR translat* OR exchange OR mobilisation OR mobilization OR disseminat* OR linkage OR management OR sharing OR share OR utilization OR utilisation OR distribut* OR diffus* OR realization OR realisation OR embodiment OR implement* OR uptake OR "to action" OR "to practice" OR "into practice")) |
| S4 | (tiab(health* OR wellbeing OR well-being OR recover* OR resilien* OR wellness OR heal OR healing OR trauma-informed OR "harm reduction") OR tiab(clinic* OR hospital* OR ((health OR treatment OR care) NEAR/2 (center* OR centre*)))) |
| S5 | [S2] AND [S3] AND [S4] AND [S5] |
| S6 | ([S2] AND [S3] AND [S4] AND [S5]) AND yr(2010-2024) |
